# Anti-Inflammatory and Antinociceptive Effects of Ethyl Acetate Fraction of an Edible Red Macroalgae *Sarcodia ceylanica*

**DOI:** 10.3390/ijms18112437

**Published:** 2017-11-17

**Authors:** Chieh-Chih Shih, Hwong-Ru Hwang, Chi-I Chang, Huei-Meei Su, Pei-Chin Chen, Hsiao-Mei Kuo, Pei-Jyuan Li, Hui-Min David Wang, Kuan-Hao Tsui, Yu-Chi Lin, Shi-Ying Huang, Zhi-Hong Wen

**Affiliations:** 1 Department of Marine Biotechnology and Resources, National Sun Yat-sen University, Kaohsiung 80424, Taiwan; shih.chiehchih@gmail.com; 2 Department of Marketing and Distribution Management, Fortune Institute of Technology, Kaohsiung 83158, Taiwan; 3 Division of Cardiology, Department of Internal Medicine, Pingtung Christian Hospital, Pingtung 90059, Taiwan; hwang.lin@msa.hinet.net; 4 Division of Cardiology, Department of Internal Medicine, Kaohsiung Veterans General Hospital, Kaohsiung 81362, Taiwan; 5 Department of Biological Science and Technology, National Pingtung University of Science and Technology, Pingtung 91201, Taiwan; changchii@mail.npust.edu.tw; 6 Tungkang Biotechnology Research Center, Fisheries Research Institute, Council of Agriculture, Pingtung 92845, Taiwan; healthalgae@gmail.com; 7 Doctoral Degree Program in Marine Biotechnology, National Sun Yat-sen University and Academia Sinica, Kaohsiung 80424, Taiwan; peichin1128@gmail.com; 8 Center for Neuroscience, National Sun Yat-sen University, Kaohsiung 80424, Taiwan; hsiaomeikuo@gmail.com; 9 Marine Biomedical Laboratory and Center for Translational Biopharmaceuticals, Department of Marine Biotechnology and Resources, National Sun Yat-sen University, Kaohsiung 80424, Taiwan; pecha.pipi@gmail.com; 10 Graduate Institute of Biomedical Engineering, National Chung Hsing University, Taichung 40227, Taiwan; davidw@dragon.nchu.edu.tw; 11 College of Oceanology and Food Science, Quanzhou Normal University, Quanzhou 362000, China; 12 Department of Obstetrics and Gynecology, Kaohsiung Veterans General Hospital, Kaohsiung 81362, Taiwan; khtsui60@gmail.com; 13 Department of Obstetrics and Gynecology and Institute of Clinical Medicine, National Yang-Ming University, Taipei 11221, Taiwan; 14 Department of Pharmacy and Graduate Institute of Pharmaceutical Technology, Tajen University, Pingtung 90741, Taiwan; 15 Division of Chinese Materia Medica Development, National Research Institute of Chinese Medicine, Taipei 112, Taiwan; m8952612@hotmail.com; 16 Fujian Province Key Laboratory for the Development of Bioactive Material from Marine Algae, Quanzhou 362000, China; 17 Key Laboratory of Inshore Resources Biotechnology (Quanzhou Normal University), Fujian Province University, Quanzhou 362000, China

**Keywords:** *Sarcodia ceylanica*, carrageenan, pain, leukocyte infiltration, oral

## Abstract

Research so far has only shown that edible red macroalgae, *Sarcodia ceylanica* has the ability to eliminate free radicals and anti-diabetic, anti-bacterial properties. This study was conducted both in vitro and in vivo on the ethyl acetate extract (PD1) of farmed red macroalgae in order to explore its anti-inflammatory properties. In order to study the in vitro anti-inflammatory effects of PD1, we used lipopolysaccharide (LPS) to induce inflammatory responses in murine macrophages. For evaluating the potential in vivo anti-inflammatory and antinociceptive effects of PD1, we used carrageenan-induced rat paw edema to produce inflammatory pain. The in vitro results indicated that PD1 inhibited the LPS-induced pro-inflammatory protein, inducible nitric oxide synthase (iNOS) in macrophages. Oral PD1 can reduce carrageenan-induced paw edema and inflammatory nociception. PD1 can significantly inhibit carrageenan-induced leukocyte infiltration, as well as the protein expression of inflammatory mediators (iNOS, interleukin-1β, and myeloperoxidase) in inflammatory tissue. The above results indicated that PD1 has great potential to be turned into a functional food or used in the development of new anti-inflammatory and antinociceptive agents. The results from this study are expected to help scientists in the continued development of *Sarcodia ceylanica* for other biomedical applications.

## 1. Introduction

The development of marine natural products has seen substantial growth and as many as the biological activities of 4196 compounds have been confirmed. Algae accounts for 25% of marine natural compounds while the remainder come from coelenterata and echinodermata [1]. The 25% of development in the marine natural product field is in algae products, and the key reasons are that algae are regenerative, edible and environmentally friendly. Compared to green and brown algae, red algae biodiversity is higher, which allows for more active substances to be developed. For example, previous papers have also found that the galactose found in *Gelidium crinale* and the polysaccharide sulfate from *Gracilaria cornea* have anti-nociceptive properties [2]. In summary, past literature has indicated that red algae have great potential with respect to the development of new drugs.

*Sarcodia ceylanica* was originally a traditional delicacy on Taiwan’s Liuqiu Island. Over-harvesting in recent years had decreased its numbers and threatened its survival, and to this end, the Fisheries Research Institute (FRI) of the Council of Agriculture (COA) announced in October 2012 that it had successfully developed an artificial breeding technique for *Sarcodia ceylanica*. There are barely any existing studies on the biological activity of farmed *Sarcodia ceylanica*. To our knowledge, only two non-SCI journal papers have been written regarding *Sarcodia ceylanica*’s bioactive properties. The research results showed that the water extract from *Sarcodia ceylanica* can eliminate free radicals, possesses anti-diabetic properties, and reduces triglyceride levels in rats [3]. The phosphate buffered saline (PBS) extracted from *Sarcodia ceylanica* was also discovered to have anti-bacterial activity [4]. In vivo test results have also demonstrated that the water extract from the same layer of a different species of macroalgae, *Sarcodia ceylonensis*, has demonstrated anti-tumor activities, as well as immune regulation properties [5]. *Sarcodia ceylonensis*’s water extract was also found to have an anti-oxidative effects [6]. Based on the previous studies, not much is known about the active ingredients of *Sarcodia ceylanica* compared to other red algae. In the field of marine natural products for biomedical applications, several studies have previously been successful in identifying biological activities from extract and fraction of the cultivated strains of the red seaweed, such as *Chondrus crispus* [7]. Thus, present study employed in vitro and in vivo tests to explore the anti-inflammatory and antinociceptive effects of the ethyl acetate extract PD1 in *Sarcodia ceylanica*.

The inflammatory cells release various pro-inflammatory mediators such as inducible NOS (iNOS) and interleukin-1β (IL-1β). IL-1β activates pain sensory nerves and transmits to the brain, thereby causing an organism to be affected by the pathological conditions that is nociception [8]. The iNOS is primarily expressed in macrophages. It is expressed at a high level when the macrophages are activated during an inflammatory response, thus accelerating the biosynthesis of nitric oxide (NO), which may ultimately cause cytotoxicity, septic shock, cancer and other serious medical conditions [9]. The IL-1β is the strongest pro-inflammatory mediator in the interleukin family. Previous studies have shown that injection of IL-1β or iNOS inhibitors can attenuated nociception in neuropathic rats [10]. The above literatures show that both iNOS and IL-1β play an important role in the inflammatory responses, thus the ability to inhibit them effectively would greatly aid the development of new anti-inflammatory drugs. When cells experience a level of oxidative stress higher than that of the anti-oxidative effect, the synthesis and release of inflammatory factors is further promoted [11]. Myeloperoxidase (MPO) often serves as a marker of oxidative stress, for it can be used to evaluate the inhibitory effect of substances on oxidative stress [12]. In damaged tissues, the effect of a substance in reducing MPO levels is reflective of its ability to reduce oxidative stress. Moreover, MPO is also a leukocyte marker [13,14], hence MPO levels can serve as an indicator when analyzing the extent of tissue inflammation. Furthermore, NO can autoxidize to nitrite (NO_2_^−^), and MPO uses NO_2_^−^ to generate bactericidal oxidants [15]. Eiserich et al. demonstrated that formation of NO-derived inflammatory oxidants by MPO in neutrophils [16]. Additionally, the several previous studies also showed that the link between NO pathway and MPO [17,18,19].

In order to study the anti-inflammatory effects of the ethyl acetate extract PD1 from *Sarcodia ceylanica*, lipopolysaccharide (LPS) was used in vitro tests to induce inflammatory responses in murine macrophages. For the in vivo tests, we used carrageenan-induced rat paw edema, which produced inflammatory pain, and evaluated the protein levels of iNOS, IL-1β and MPO to study the potential anti-inflammatory and antinociceptive effects of PD1.

## 2. Results

### 2.1. Cell Viability and Anti-Inflammatory Effects of PD1 with Respect to LPS-Induced iNOS and COX-2 in RAW 264.7 Cells

**Figure 1:**
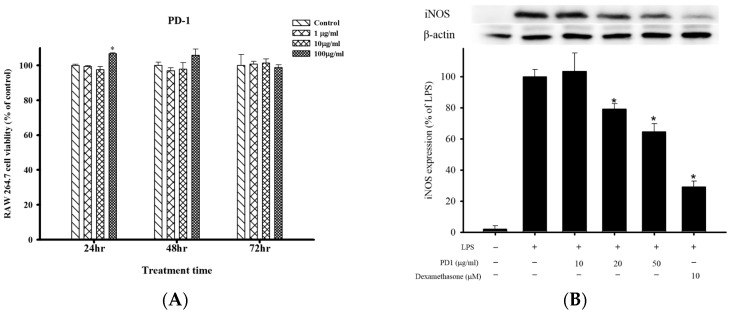

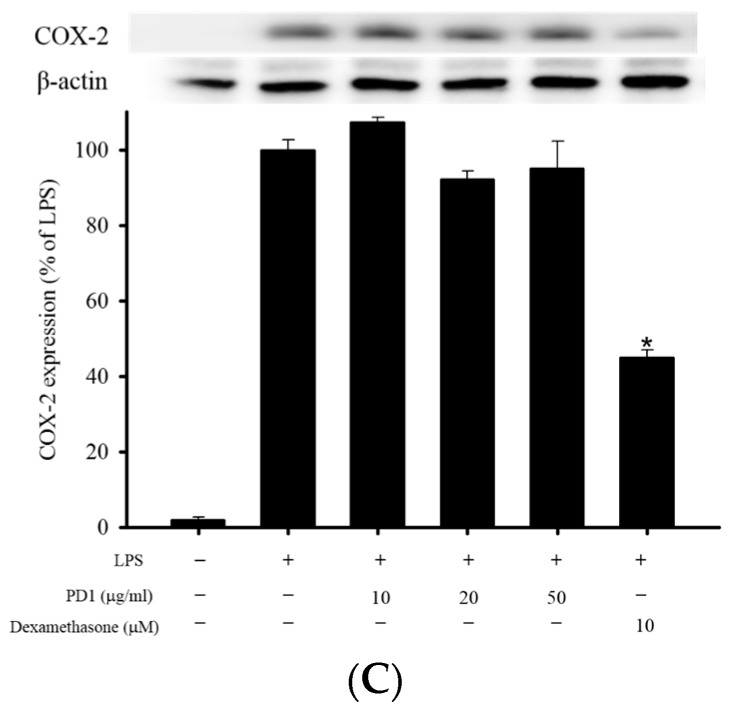
The effects of different concentrations of ethyl acetate extract (PD1) on the cell viability of RAW 264.7. (**A**) Use of Almar Blue assay to analyze the effects of PD1 on the cell viability of RAW 264.7 after 24, 48 and 72 h. The horizontal axis shows the time in h from vehicle or PD1 treatment, and the vertical axis shows the percentage of the cell viability of RAW 264.7. Data presentation: mean ± SEM; * *p <* 0.05, compared to the control group. Effects of PD1 on the LPS-induced inflammatory protein iNOS (**B**) and COX-2 (**C**) in RAW 264.7. In figures (**B**,**C**), the horizontal axis shows the treatment groups, and the vertical axis shows the levels of the protein expression. Data presentation: mean ± standard error of the mean (SEM); * *p <* 0.05, compared to the lipopolysaccharide (LPS)-only group.

Using Almar Blue reduction assay, we analyzed the effects of PD1 on the cell viability of RAW 264.7 after 24, 48 and 72 h (Figure 1A). The results showed that PD1 (1 μg/mL, 10 μg/mL) did not significantly affect the cell viability of RAW 264.7 after 24, 48 and 72 h. At 24 h, cell viability had increased significantly with PD1 (100 μg/mL) compared to the control group. The test results indicated that, other than a significant rise in RAW 264.7 cell viability 24 h after PD1 (100 μg/mL) was administered, the remaining permutations did not significantly affect cell viability. Protein expression of iNOS in the LPS-only group was 100%. After the administration of 10 μg/mL, 20 μg/mL and 50 μg/mL of PD1, iNOS protein expression became 103.1 ± 11.6%, 79.1 ± 3.4% and 64.6 ± 5.0% respectively. Statistical analysis revealed that PD1 at the 20 μg/mL and 50 μg/mL can significantly inhibit the protein expression of LPS-induced iNOS in RAW 264.7 cells (Figure 1B). In addition, in the positive control group in which dexamethasone was added, iNOS expression was significantly inhibited when compared to the LPS group. The protein expression of cyclooxygenase-2 (COX-2) was 100% in the LPS-only group. After the administration of 10 μg/mL, 20 μg/mL and 50 μg/mL of PD1, statistical analysis showed that the LPS-induced COX-2 protein expression in RAW 264.7 cells was not significantly inhibited by PD1 (Figure 1C). We also observed that PD1 resulted in no significant difference for β-actin.

### 2.2. Effects of Oral PD1 on Carrageenan-Induced Rat Paw Edema

**Figure 2:**
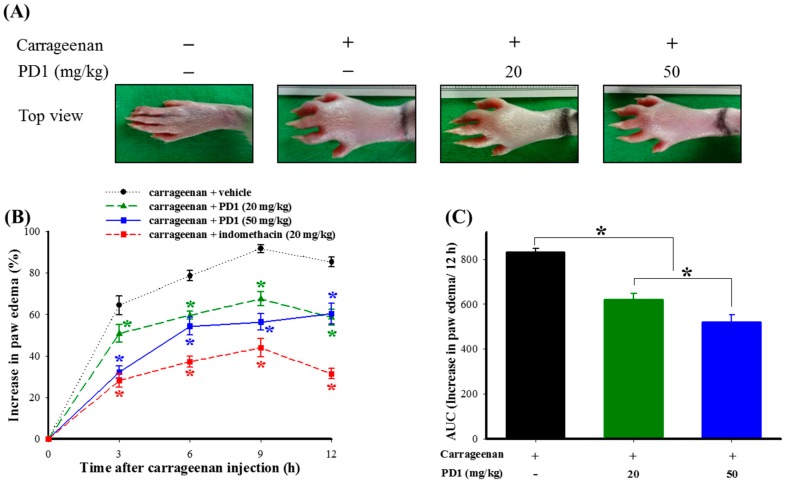
Effects of PD1 on carrageenan-induced rat paw edema: (**A**) The time course of the percentage change of the increase in paw volume induced by carrageenan in rats treated with PD1; (**B**) The horizontal axis shows the time in h from carrageenan injection, and the vertical axis shows percentage change of increased paw volume. The different concentrations of PD1 and the extent of rat paw edema was plotted along a time curve and converted into AUC scores; (**C**) The horizontal axis shows the treatment groups, and the vertical axis shows the area under the paw edema effect–time curve. Compared to the carrageenan + vehicle group, a smaller value means that PD1 has a stronger inhibitory effect on carrageenan-induced rat edema. We used 20 mg/kg indomethacin as a positive control. Data presentation: mean ± SEM with 6 rats per group; * *p <* 0.05, compared to the carrageenan + vehicle group.

In vivo anti-inflammatory testing involving use of PD1 on carrageenan-induced rat paw edema. Pathological phenomena observed on rat paws were photographed (Figure 2A), and the photographical results showed that the rat’s paw underwent significant swelling after the injection of carrageenan. Oral PD1 (20 or 50 mg/kg), the rat paw edema was suppressed. Concurrently, we used a paw volume meter to measure changes in rat paw edema (Figure 2B) and found that the carrageenan-induced rat paw edema was most significant during the 9th hour, following which the swelling gradually subsided. Rats that also received PD1 (20 or 50 mg/kg) saw significant suppression of the carrageenan-induced paw edema. In addition, in the positive control group in which indomethacin (20 mg/kg) was added, there was also a reduction in rat paw edema compared to the carrageenan + vehicle group. The extent of rat paw edema was plotted along a time curve and converted into the area under the curve (AUC) scores (Figure 2C). The results showed that administering PD1 (20 or 50 mg/kg) led to a significant reduction of paw edema when compared to the carrageenan + vehicle group.

### 2.3. Effects of PD1 on Carrageenan-Induced Inflammatory Nociceptions

**Figure 3:**
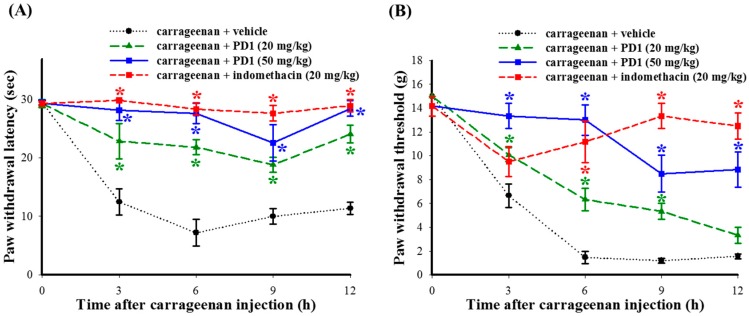
Time-courses of antinociceptive effects of oral PD1 or indomethacin on carrageenan-induced nociceptive behaviors, including thermal hyperalgesia (**A**) and mechanical allodynia (**B**). We used 20 mg/kg indomethacin as a positive control. Data are the mean ± SEM with 6 rats per group; * *p <* 0.05, compared to the carrageenan + vehicle group.

The use of carrageenan to induce thermal hyperalgesia and mechanical allodynia in rats for the purpose of assessing the antinociceptive effects of PD1 on inflammatory pain. Our results showed that carrageenan-induced thermal hyperalgesia and mechanical allodynia will persist for 12 h or more. The administering of PD1 (20 or 50 mg/kg) significantly improved thermal hyperalgesia (Figure 3A) and mechanical allodynia (Figure 3B) conditions induced by carrageenan, with said conditions persisting for 12 h. A group given indomethacin (20 mg/kg) acted as the positive control group. When compared to the carrageenan + vehicle group, it also showed an improvement with respect to the thermal hyperalgesia and mechanical allodynia phenomena in rats.

### 2.4. Histopathologic Analyses of the Effects of PD1 on Cell Infiltration in Carrageen-Injected Paws

**Figure 4:**
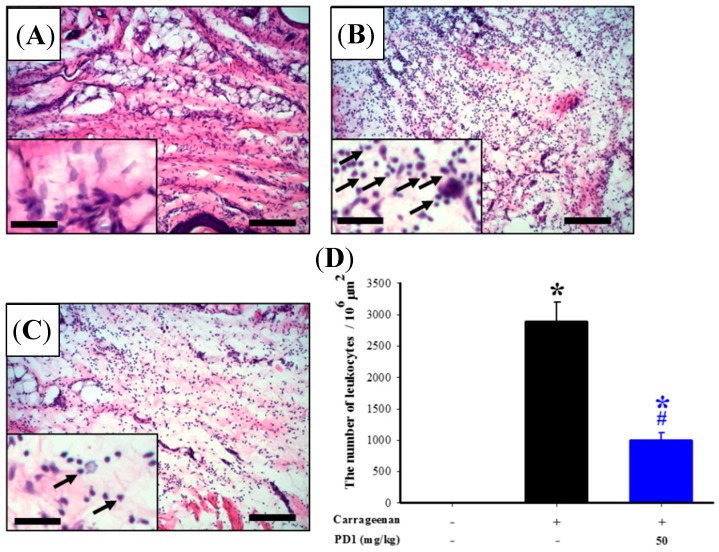
Effect of PD1 on changes to carrageenan-induced leukocyte infiltration in rat paw tissue. The figure above shows the changes to leukocyte infiltration in rat paw tissue from control (**A**); carrageenan (**B**); carrageenan + PD1 (**C**) groups, as well as quantification (**D**) of the number of leukocytes (arrows). In the figure (**D**), the horizontal axis shows the treatment groups, and the vertical axis shows the leukocyte numbers in rat paw tissues. Scale bars: (**A**–**C**) 150 μm; (**A**) inset, (**B**) inset and (**C**) inset, 35 μm. Data are mean ± SEM with 6 rats per group; * *p <* 0.05, compared to the control group; # *p <* 0.05, compared to the carrageenan group.

The hematoxylin-eosin stain method was used to carry out histopathologic analysis on rat paw biopsy tissue. The hematoxylin and eosin (H&E) staining results showed that, when compared to the control group (Figure 4A), carrageenan promotes leukocyte infiltration in rat paw tissues (Figure 4B). The administration of PD1 can reduce the level of carrageenan-induced leukocyte infiltration (Figure 4C). Statistical results (Figure 4D) also showed that the oral ingestion of PD1 can reduce the extent of the carrageenan-induced increase in leukocyte numbers.

### 2.5. Effects of PD1 on Inflammatory-Related Protein Expression in Rat Paw Tissues Induced by Carrageenan

**Figure 5:**
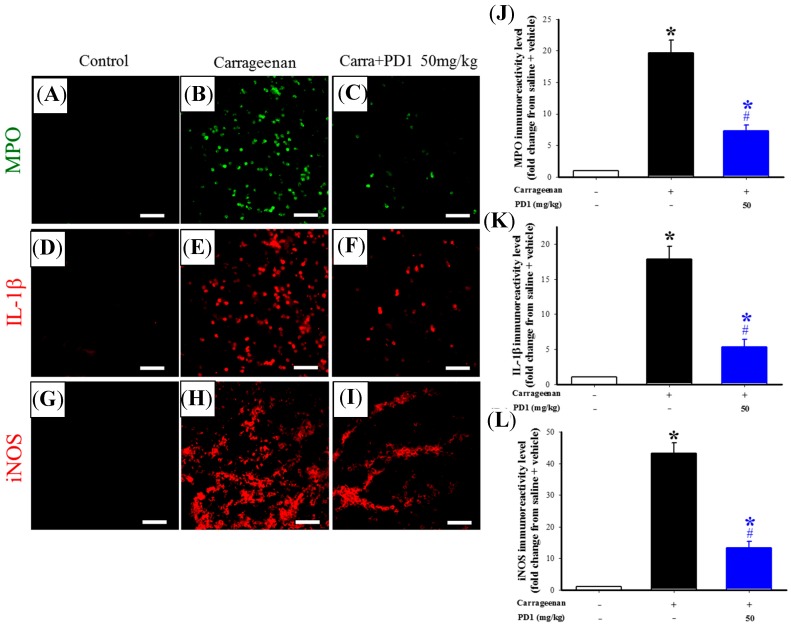
Effects of PD1 on the carrageenan-induced upregulation of immunoreactivity of MPO (green), IL-1β (red) and iNOS (red) in rat paw tissue. Control (**A**,**D**,**G**), carrageenan (**B**,**E**,**H**), and carrageenan + PD1 (**C**,**F**,**I**) groups. Immunoreactivity quantitative results of MPO (**J**), IL-1β (**K**) and iNOS (**L**). In the quantitative figures (**J**,**K**,**L**), the horizontal axis shows the treatment groups, and the vertical axis shows the levels of immunoreactivity of the protein. Data are mean ± SEM with 6 rats per group; * *p <* 0.05, compared to the control group; # *p <* 0.05, compared to the carrageenan group; scale bar: 50 μm.

The immunohistochemistry method was used to analyze the effects of PD1 on the carrageenan-induced protein expressions of MPO, IL-1β and iNOS in rat paw tissue. The results showed that the immunoreactivity of carrageenan-induced MPO, IL-1β and iNOS in rat paw tissue (Figure 5B,E,H) increased significantly when compared to the control group (Figure 5A,D,G). After PD1 treatment, the immunoreactivity of MPO, IL-1β and iNOS showed a significant decrease (Figure 5C,F,I). The fluorescence quantification results indicated that oral PD1 significantly inhibited the upregulation of immunoreactivity of MPO, IL-1β and iNOS in carrageenan-injected paw tissue (Figure 5J,K,L). This indicated that PD1 inhibited carrageenan-induced inflammation in rat paw tissue.

### 2.6. Effects of PD1 on Expression of Carrageenan-Induced IL-1β and iNOS in Leukocytes in Rat Paw Tissues

**Figure 6:**
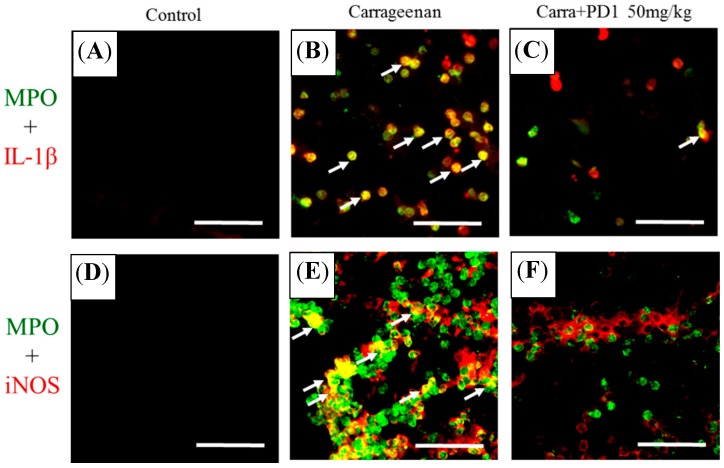
Effects of PD1 on expression of carrageenan-induced IL-1β (red) and iNOS (red) in leukocytes (MPO-positive cells; green) in rat paw tissues. The sections are show for control (**A**,**D**); carrageenan (**B**,**E**) and the carrageenan + PD1 (**C**,**F**). Double immunofluorescent staining revealed that PD1 significantly inhibited carrageenan-induced co-localization (arrows) of MPO with iNOS and IL-1β. Scale bar: 50 μm.

Double immunohistochemistry was used to observe the effects of PD1 on the expression of carrageenan-induced IL-1β and iNOS in leukocytes (MPO-positive cells) in rat paw tissues. The results showed that the immunoreactivity expression of MPO and IL-1β was extremely low in the control group (Figure 6A). In the carrageenan group (Figure 6B), we observed that IL-1β showed a high level of expression in MPO-positive cells, although such an expression was inhibited by orally ingested PD1 (Figure 6C). Also, the immunoreactivity of iNOS in MPO-positive cells in the control group was extremely low (Figure 6D). In the carrageenan group (Figure 6E), we observed a high level of expression of iNOS in MPO-positive cells, but such a phenomenon can be inhibited by orally ingested PD1 (Figure 6F).

## 3. Discussion

In present study, the in vitro results showed that PD1 from *Sarcodia ceylanica* was not cytotoxic at the 100 μg/mL concentration level. At 20 μg/mL, it significantly inhibited the expression of the pro-inflammatory protein iNOS. In the carrageenan-induced inflammation of paws, results showed that orally ingested PD1 alleviated nociceptive behaviors and paw swelling. PD1 inhibited the inflammatory responses resulting from carrageenan-induced leukocyte infiltration within paw tissues, and also reduce the expression of inflammatory mediators such as MPO, iNOS and IL-1β. No significant abnormalities were observed with respect to the appearance or behavior of rats that orally ingested PD1. Our results showed that the PD1 has anti-inflammatory and antinociceptive properties.

Overactivation of iNOS leads to a massive amount of NO being produced, which will further exacerbate inflammation in tissues [20]. It may even result in a range of chronic diseases such as: cancer, diabetes, and cardiovascular diseases [21]. For the screening and selection of bioactive substances with anti-inflammatory properties, many research reports used an in vitro model in which LPS-induced mouse macrophage cell lines (RAW 264.7) are utilized to produce inflammation responses [22,23]. We have previously been successful in using this model to screen and select marine compounds with anti-inflammatory properties [24,25]. The in vitro experimental results showed that PD1 can significantly inhibit the upregulation of the pro-inflammatory protein iNOS caused by LPS challenged mouse macrophages (RAW 264.7 cells), and PD1 was also shown to have a dose-dependent effect in this case (Figure 1B). However, it had no significant effect on COX-2 (Figure 1C). Previous studies indicated that iNOS is involved in the regulation of COX-2 and plays a key role in inflammatory factors [24]. Based on our results, we suggest that the PD1-induced anti-inflammatory effects observed occur via attenuation of iNOS protein expression, but not via COX-2 protein expression level.

Among existing studies assessing the in vivo anti-inflammatory and anti-nociceptive activity of natural products, carrageenan-induced rat paw edema is the most widely used model for research into inflammation [26]. We used carrageenan to induce inflammatory phenomena in rat paws, so as to assess the in vivo anti-inflammatory and anti-nociceptive effects of PD1. The results show that orally ingesting PD1 can reduce carrageenan-induced swelling (Figure 2B) in paws, as well as pain behaviors such as thermal hyperalgesia (Figure 3A) and mechanical allodynia (Figure 3B). Thus, based on the results of inhibition of carrageenan-induced nociception, we concluded PD1 effectively reduced inflammatory pain in carrageenan-injected rats model.

Carrageenan is injected into rat paws, the damaged tissues result in mast cells becoming stimulated and releasing bradykinin, serotonin and histamine to raise vascular permeability. This causes a rat paw to gradually swell, leading to a phagocytic inflammatory response. Moreover, the damaged tissues will then release cytokines, which will promote leukocytes to participate in the phagocytic reaction and promotes the expression of iNOS, thus leading to the production of a large number of pro-inflammatory mediators (e.g., prostaglandin), and eventually, inflammatory pain [27]. Previous research has proven that, after injecting carrageenan into a rat’s paw to induce paw tissue inflammation, leukocytes will gather at the inflamed area, which in turn induces infiltration in cells [28]. We also discovered in our previous research that carrageenan can induce a high level of leukocyte infiltration in rat paw tissues [29]. In this study, we used H&E stain to analyze biopsy tissue and observed that the inflamed tissues were affected by leukocyte infiltration, which was inhibited by orally ingested PD1 (Figure 4). We have also observed, through immunofluorescence, that carrageenan will induce the expression of massive amounts of pro-inflammatory proteins like iNOS, IL-1β and MPO in rat paw tissues, and this phenomenon was inhibited by orally ingested PD1 (Figure 5). Existing studies have already shown that iNOS is the most important regulating enzyme in inflammatory responses. When iNOS is overexpressed and causes a massive amount of NO to be produced, severe inflammation and eventually diseases would occur [9]. Similar to previous studies [24,27], we discovered a massive amount of iNOS protein being expressed in the rat paws from the carrageenan group. Past research has shown that iNOS plays an important pathological role in carrageenan-induced inflammatory responses [24,29]. Systemic administration of selective iNOS inhibitors can inhibit carrageenan-induced paw swelling [27] and pain behavior [26]. In addition to being a pro-inflammatory mediator, IL-1β can also regulate immune functions. Injecting a rat with interleukin-1 receptor antagonist can reduce the production of neuropathic pain in it [10]. MPO also possesses pro-inflammatory properties [30]. Based on the above literatures, iNOS, IL-1β and MPO all play important roles in inflammatory responses, and we discovered that PD1 can inhibit the expression of these three pro-inflammatory proteins.

MPO is primarily present in the primary granules of leukocytes and responsible for primary immunity [31]. When bacteria invade an organism, leukocytes will engulf them via phagocytosis and form a phagosome around them where activation and degranulation will then occur. At the same time, MPO will be released from the primary granules into the phagosome and cells. It is then catalyzed to produce hypochlorous acid (HOCl), tyrosyl radical and reactive oxidative species, these are oxidative agents that can kill invading bacteria effectively [32]. By measuring MPO expression, we can therefore assess the extent of oxidative damage [12]. In addition, MPO can also promote inflammation and atherosclerosis. This can affect the stability of plaque, which will in turn lead to acute coronary syndromes (ACS). Thus, MPO can also be used as an inflammatory marker to predict ACS [30]. By reducing MPO expression in damaged tissues, we can help reduce the occurrence of inflammation and oxidative stress. In this study, we discovered that orally ingested PD1 can slow down MPO production in carrageenan-induced inflammatory rats. In other words, we proposed that PD1 can also potentially be applied in the field of cardiovascular health. Moreover, it has been that known that MPO is also a leukocyte marker [13,14]. In addition, given that we have found a significant increase in the expression of MPO immunoreactivity in rat paw tissue from the carrageenan group, this also proves that leukocyte infiltration had indeed occurred (Figure 4). In the rat paws from the carrageenan group, we observed a massive amount of iNOS and IL-1β being expressed, and co-localization with MPO occurred for both proteins (Figure 6B,E). They were inhibited by orally ingested PD1 (Figure 6C,F). The anti-inflammatory effects of PD1 have been associated with both inhibiting leukocyte infiltration and downregulating pro-inflammatory protein iNOS and IL-1β expression in MPO-positive cells, which participate in lowering the level of local tissue inflammation.

Whether a marine natural product can be supplied in a sustained and stable manner is often one of the key determining factors in its chances of undergoing preclinical and clinical trials [33,34]. Since *Sarcodia ceylanica* can be farmed, we have a steady supply of PD1. Based on these research results, the advantages of PD1 include the fact that it can be orally ingested, its low cytotoxicity, and its anti-inflammatory and antinociceptive properties. At the same time, rats that orally ingested PD1 did not exhibit any significant abnormal motor behavior or abnormalities in our experiment. In the previous research on the biological activity of extracts from *Sarcodia ceylanica* [3] or *Sarcodia ceylonensis* [5,6], its primary bioactive ingredient was polysaccharide. The extraction method used in this study is different from most other polysaccharide extraction methods [35,36]. A commonly used polysaccharide extraction method is to dip and stir dry algae powder for 72 h in a 0.15 N HCl solution. After obtaining the clarified liquid from the centrifuge, ethanol is added and stirring is carried out for 24 h to allow for polysaccharide precipitation, finally polysaccharide is obtained after the solution is dried through vacuum cooling. By using the ethanol extraction method for macroalgae in present study, we ensured that the polysaccharides were not dissolved, and that the ethanol extract did not contain any polysaccharides. At the same time, our negative phenol-sulfuric acid test results also showed that PD1 does not contain polysaccharides with the method described in previous study [35,36]. The future development of PD1 will move in three directions. Firstly, the identification of the possible compound structures in PD1 to mark active ingredients and facilitate the future development of related drugs. In addition, the high performance liquid chromatography (HPLC) profile of PD1 (Figure S1) from this study is expected to help scientists in the continued investigation of the information regarding the chemical composition of PD1. Until now, the chemical constituents of *Sarcodia ceylanica* are still unclear. Basing on our preliminary isolation results and the UV-vis absorption bands of peak of HPLC chromatogram (Table S1), we tendentiously predicted that peaks 6 and 8 would belong to the acylglycerol compounds (λ_max_ 220 nm) with an unsaturated fatty acid moiety [37], peak 2 would be an aromatic compound (λ_max_ 256 nm), and other peaks (1, 3, 4, 5, and 7) would be also aromatic compounds with an additional conjugated carbonyl group (λ_max_ 270–292 nm). The spectra of the molecules coupling with our preliminary isolation results, the glycerol and benzene derivatives are the major constituents in PD1. Patients suffering from chronic pain have to endure the serious side effects often associated with the long-term use of pain medications, or worry about addiction problems. Anti-inflammatory dietary supplements that are highly safe and can be taken long-term have become a new field of development for pain relief [38]. Several studies have previously been successful in identifying bioactive constituents from foods, including anti-inflammatory [39,40] and antinociceptive [41,42] components. *Sarcodia ceylanica* was originally a general food product, and through our research, we have found that its extract PD1 has anti-inflammatory and antinociceptive properties, which are qualities in line with those of anti-inflammatory dietary supplements. The second direction would be to continue research into the cellular mechanisms of PD1 anti-inflammatory and antinociceptive properties, and to explore the efficacy of PD1 with respect to other inflammatory pain disorders, such as arthritis, with the aim of developing PD1 into a functional food. The final direction would be to explore other potential biological activities in PD1. The results from this study are expected to help scientists in the continued development of *Sarcodia ceylanica* for other biomedical applications.

## 4. Materials and Methods

### 4.1. Materials

The farmed *Sarcodia ceylanica* used in this study came from the Fisheries Research Institute (FRI), Taiwan.

### 4.2. Preparation Method for Crude Extract

We dried the *Sarcodia ceylanica* at 50 °C for 24 h. The dried *Sarcodia ceylanica* was crushed into powder and then soaked in ethanol (95%) at room temperature for extraction. The soaking solution was filtered and concentrated under reduced pressure to obtain the crude ethanol extract (34.3 g from 1 kg dried weight), which was suspended in H_2_O (1 L) and partitioned using 1 L of EtOAc. This partition process was carried out thrice to obtain 7.7 g of the abovementioned ethyl acetate fraction (PD1). At the same time, we also used the phenol-sulfuric acid method to measure the polysaccharide content of PD1. Our measurements showed that PD1 did not contain polysaccharide [35,36].

### 4.3. The Preparation of Analytical Sample and HPLC Conditions

10 mg of PD1 of Sarcodia ceylanica was dissolved in 1 ml of dimethyl sulfoxide (DMSO) and was filtered through a 0.22 μm micropore membrane prior to analysis. Analytical HPLC analysis was performed using a Hitachi L-7000 chromatograph. Separation of PD1 was carried out at 25 °C using a 250 × 4.6 mm i.d., 5 μm, BDS Hypersil C18 column from Thermo Electron Corporation (Waltham, MA, USA). The mobile phase consisted of (A) water containing 0.5% acetic acid and (B) acetonitrile. The program for gradient elution started at 95% solvent A and 5% solvent B, increased linearly to 50% solvent A and 50% solvent B in 50 min. The mobile phase was pumped at the flow rate of 1.0 mL/min with 20 μL injection volume. The UV absorbance detection wavelength was set at 256 nm.

### 4.4. Cell Culture

#### 4.4.1. Preparation of Cell

With modified method from previous studies [22,23,29,43], we assessed anti-inflammatory activity of PD1. We obtained murine RAW 264.7 macrophages cells from the American Type Culture Collection (TIB-71; Manassas, VA, USA), and cultured macrophages cells in Dulbecco’s modified Eagle’s medium supplemented with 10% heat-inactivated fetal bovine serum, 50 U/mL penicillin, 50 μg/mL streptomycin, 2 mM glutamine, 1 mM pyruvate, and 4.5 g/L glucose, at 37 °C in a humidified incubator (5% CO_2_:95% air).

#### 4.4.2. Cell Viability

We determined cell viability after treatment with Alamar Blue (Invitrogen, Carlsbad, CA, USA), the tetrazolium dye that is reduced by living cells to fluorescent products, which is similar in principle to the cell viability assay using 3-(4,5-dimethyldiazol-2-yl)-2,5-diphenyltetrazolium bromide. This method has been validated as an accurate measurement for the survival of RAW 264.7 macrophage cells [44]. We dissolved PD1 in 100% dimethyl sulfoxide (DMSO) in cell culture experiments. In the final culture medium, the concentration of DMSO was 1%.

### 4.5. In Vitro Anti-Inflammatory Assay

We induced inflammation in murine RAW 264.7 macrophages by incubating macrophages in a medium containing only LPS (0.01 μg/mL; L2654; Sigma, St. Louis, MO, USA) for 16 h. For anti-inflammatory activity assay, 5 min before LPS challenge, we added PD1 (10, 20 or 50 μg/mL) or positive control (dexamethasone, 10 μM) to macrophages. Then, to wash macrophages with ice-cold phosphate-buffered saline (PBS), lysed macrophages in ice-cold lysis buffer, and then centrifuged macrophages at 20,000× *g* for 30 min at 4 °C. We decanted and retained the supernatant from the pellet, and determined protein concentrations by the DC protein assay kit (Bio-Rad, Hercules, CA, USA) from a modified method of Lowry et al. We performed Western blotting with the method described in previous study [29,45]. To add an equal volume of sample buffer into the sample. To load sample onto a tricine SDS-polyacrylamide gel and to electrophorese sample at 150 V for 90 min. To transfer the sample into a polyvinylidene difluoride membrane (Immobilon-P, Millipore Corp., Billerica, MA, USA) at 125 mA overnight at 4 °C in transfer buffer. To use 5% non-fat dry milk to block the membrane for 50 min at room temperature, and then to incubate the membrane with antibodies against iNOS (1:1000 dilution; catalog no. 6103322; polyclonal antibody; BD Pharmingen, San Diego, CA, USA) and COX-2 (1:1000 dilution; catalog no. 160106; polyclonal antibody; Cayman Chemical, Ann Arbor, MI, USA) proteins for 180 min at room temperature. We utilized chemiluminescence (Millipore Corp.) for detecting a horseradish peroxidase-conjugated secondary antibody. With the UVP BioChemi Imaging System and LabWorks 4.0 software (UVP, Upland, CA, USA), we obtained images for relative densitometric quantification. We calculated relative variations between the bands of the PD1- or compound-treatment samples and the only LPS-treatment samples with the same image.

### 4.6. In Vivo Model for Carrageenan-Induced Rat Paw Inflammation

With the Guiding Principles in the Care and Use of Animals of the American Physiology Society, we maintained Wistar rats (weight: 250–285 g; LASCO, Taipei, Taiwan) in a temperature-controlled (22 ± 1 °C) and 12-h light-dark cycle room, which were approved by Animal Care and Use Committee of National Sun Yat-sen University Kaohsiung, Taiwan (10408). After rats were anesthetized with 4% isoflurane in a plastic box in room air, we delivered 2.5% isoflurane in an air/O_2_ mixture to rats with a mask. We used the rat inflammatory model with a modification of the model of Winter et al. as previously described in previous study [24,29,43]. An hour before intraplantar injection into its right hind paw with 1.5% sterile carrageenan lambda (Sigma) in 100 μL of saline, a rat was orally fed a 2 mL mix containing solvent blank (vehicle; 2% DMSO), PD1 (20 or 50 mg/kg), or indomethacin (20 mg/kg; Cayman Chemical Co., Ann Arbor, MI, USA).

### 4.7. Analyses of the Edema and Pain Behavior of Rats

Using a method described in our previous reports [29,46], we used a paw volume meter (plethysmometer; Singa Technology Corporation, Taipei, Taiwan) to measure changes in rat paw edema. For assessing mechanical allodynia from measuring hind paw withdrawal thresholds (g), to place rats on top of an elevated metal mesh floor. As described previously by previous study [29,43], to apply a series of von Frey filaments (Stoelting, Wood Dale, IL, USA) to the mid-plantar region of the paw using Chaplan’s “up-down” method for determining the closest filament to the threshold of pain response (withdrawal or licking). With an analgesiometer (low-intensity heat (active intensity = 25) with a cutoff time of 30 s; IITC, Woodland Hills, CA, USA), we evaluated thermal hyperalgesia with placing the paw on a radiant heat source for measuring paw withdrawal latency (s). We assessed paw withdrawal latency as described previously by method of Hargreaves et al. in previous studies [29,43].

### 4.8. Histopathology and Immunohistochemistry of Paw Tissues

Using a modified method used in previous studies for histopathological examination [24,29,43], we perfused intracardially rats with cold PBS containing heparin (0.2 U/mL) followed by 4% paraformaldehyde. We fixed paw samples by 4% paraformaldehyde for 2 h. We mounted tissues from the different rat groups into the same optimal cutting temperature compound block, and sectioned tissues together with microtome (Microm HM 550; Thermo Scientific, Waltham, MA, USA), and stained sections (20 μm) using hematoxylin and eosin (H&E). For immunohistofluorescence analysis, we permeabilized sections with 0.1% Triton X-100 for 20 min, incubated sections with 4% normal horse serum for 30 min, and then incubated sections with a mixture of anti-MPO (1:200 dilution; catalog no. ab9535; polyclonal rabbit; Abcam, Cambridge, UK) and anti-iNOS (1:200 dilution; catalog no. sc-7271; monoclonal mouse; Santa Cruz Biotechnology Inc., Santa Cruz, CA, USA) or anti-IL-1β (1:200 dilution; catalog no. AF-501-NA; polyclonal goat; R&D systems, Minneapolis, MN, USA) overnight at 4 °C. We incubated the sections with a mixture of Alexa Fluor 488-conjugated anti-rabbit (1:400 dilution; green fluorescence) and rhodamine-conjugated anti-mouse antibody (1:400 dilution; red fluorescence) or rhodamine-conjugated anti-goat antibody (1:400 dilution; red fluorescence) for 40 min at room temperature. Using a Leica DM-6000 CS microscope (Leica Instruments Inc., Wetzlar, Germany) with an Idea 5 MP CMOS digital camera system (SPOT Imaging Solutions, a division of Diagnostic Instruments, Inc., Sterling Heights, MI, USA) or a Xplorer Digital camera (SPOT Imaging Solutions), we analyzed all slides of the paw sections for histopathology and immunohistochemistry. With MetaMorph Imaging System software (Molecular Devices, Downington, PA, USA), we measured immunohistofluorescence data for individual pixel values of the immunoreactive-positive area by a modified method used in a previous study [47].

### 4.9. Statistical Analysis

As described previously in previous study [29,43], we provided data as the form of mean ± SEM values, analyzed data using one-way analysis of variance (ANOVA) and followed with Duncan’s method for multiple comparisons, and considered *p <* 0.05 as significant (SigmaStat v3.5; SigmaStat, San Jose, CA, USA).

## Supplementary Materials

Supplementary materials can be found at www.mdpi.com/1422-0067/18/11/2437/s1.
