# Supplementary material for: Anti-Inflammatory and Antinociceptive Effects of Ethyl Acetate Fraction of an Edible Red Macroalgae Sarcodia ceylanica
Source: Int J Mol Sci. 2017 Nov 17;18(11):2437. doi: 10.3390/ijms18112437 (PMC5713404; doi:10.3390/ijms18112437)
Supplement: Supplementary file 1 [file ijms-18-02437-s001.pdf]

International Journal of *Molecular Sciences*

MDPI

Supplementary Materials

Anti-Inflammatory and Antinociceptive Effects of Ethyl Acetate Fraction of an Edible Red Macroalgae *Sarcodia ceylanica*

Chieh-Chih Shih [1,2,†], Hwong-Ru Hwang [3,4,†], Chi-I Chang [5], Huei-Meei Su [6], Pei-Chin Chen [7], Hsiao-Mei Kuo [8], Pei-Jyuan Li [9], Hui-Min David Wang [10,11], Kuan-Hao Tsui [12,13,14], Yu-Chi Lin [15], Shi-Ying Huang [11,16,17,*] and Zhi-Hong Wen [7,9,*]

1 Department of Marine Biotechnology and Resources, National Sun Yat-sen University, Kaohsiung 80424, Taiwan; shih.chiehchih@gmail.com
2 Department of Marketing and Distribution Management, Fortune Institute of Technology, Kaohsiung 83158, Taiwan
3 Division of Cardiology, Department of Internal Medicine, Pingtung Christian Hospital, Pingtung 90059, Taiwan; hwang.lin@msa.hinet.net
4 Division of cardiology, department of internal medicine, Kaohsiung Veterans General Hospital, Kaohsiung 81362, Taiwan
5 Department of Biological Science and Technology, National Pingtung University of Science and Technology, Pingtung 91201, Taiwan; changchii@mail.npust.edu.tw
6 Tungkang Biotechnology Research Center, Fisheries Research Institute, Council of Agriculture, Pingtung 92845, Taiwan; healthalgae@gmail.com
7 Doctoral Degree Program in Marine Biotechnology, National Sun Yat-sen University and Academia Sinica, Kaohsiung 80424, Taiwan; peichin1128@gmail.com
8 Center for Neuroscience, National Sun Yat-sen University, Kaohsiung 80424, Taiwan; hsiaomeikuo@gmail.com
9 Marine Biomedical Laboratory and Center for Translational Biopharmaceuticals, Department of Marine Biotechnology and Resources, National Sun Yat-sen University, Kaohsiung 80424, Taiwan; pecha.pipi@gmail.com
10 Graduate Institute of Biomedical Engineering, National Chung Hsing University, Taichung 40227, Taiwan; davidw@dragon.nchu.edu.tw
11 College of Oceanology and Food Science, Quanzhou Normal University, Quanzhou 362000, China
12 Department of Obstetrics and Gynecology, Kaohsiung Veterans General Hospital, Kaohsiung 81362, Taiwan; khtsui60@gmail.com
13 Department of Obstetrics and Gynecology and Institute of Clinical Medicine, National Yang-Ming University, Taipei 11221, Taiwan
14 Department of Pharmacy and Graduate Institute of Pharmaceutical Technology, Tajen University, Pingtung 90741, Taiwan
15 Division of Chinese Materia Medica Development, National Research Institute of Chinese Medicine, Taipei 112, Taiwan; m8952612@hotmail.com
16 Fujian Province key laboratory for the development of bioactive material from marine algae, Quanzhou 362000, China
17 Key Laboratory of Inshore Resources Biotechnology (Quanzhou Normal University) Fujian Province University, Quanzhou 362000, China
* Correspondence: johnjohnkings@163.com or johnjohnkings@gmail.com (S.-Y.H.); wzh@mail.nsysu.edu.tw (Z.-H.W.); Tel.: +886-921253479 (S.-Y.H.); +886-7-5252021 (Z.-H.W.)
† These authors contributed equally to this work.

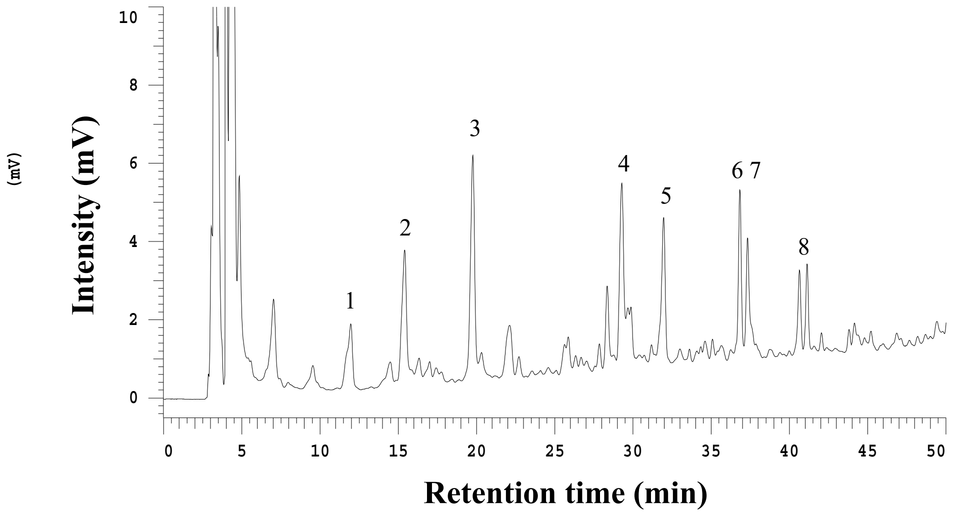

Supplementary Figure S1. HPLC profile of ethyl acetate fraction (PD1) of *Sarcodia ceylanica*. There were 8 major peaks from HPLC profile of PD1.

Supplementary Table S1. The UV absorption wavelengths of 8 peaks from HPLC profile of PD1.

Peak no.	t_R (min)	UV-vis λ_{max} (nm)
1	11.97	210, 254, 270
2	15.41	210, 256
3	19.77	211, 255, 281
4	29.29	220, 255, 289
5	31.95	222, 254, 275
6	36.83	220, 256
7	37.32	221, 254, 292
8	41.13	218, 255
